# Pedicled hand fillet flap to preserve stump length in below-elbow amputation

**DOI:** 10.1080/23320885.2022.2054420

**Published:** 2022-04-12

**Authors:** Emma Loy, David G. Williamson, J. Scott Williamson

**Affiliations:** a Island Medical Program, Faculty of Medicine, University of British Columbia, Victoria, Canada; b Department of Surgery, Faculty of Medicine, University of British Columbia, Kelowna, Canada

**Keywords:** Fillet flap, pedicled fillet-flap, spare-parts surgery, forearm amputation, below-elbow amputation

## Abstract

Spare-parts surgery in traumatic amputation sources tissue from the amputated part to cover the residual amputation defect. This case describes a trauma patient requiring below-elbow amputation. Stump closure was accomplished with a pedicled fillet flap derived from the still-attached hand, avoiding donor site morbidity and maximizing stump length.

## Introduction

Upper limb amputation is a life-altering surgery, usually carried out in adults due to trauma, infection, malignancy or vascular compromise. Many upper limb amputees use prosthetic limbs, and the level of amputation can greatly impact prosthetic functionality. Below-elbow prostheses are substantially more functional than above-elbow prostheses [1]. Therefore, a major goal of upper limb amputation is to create stable soft tissue coverage of the stump while preserving maximal stump length [1,2].

Given that the majority of upper limb amputations occur due to trauma [3], the level of amputation is often dictated by the mechanism of injury. The priority, therefore, becomes maintaining maximal bone length, rather than shortening bone to obtain primary tension-free wound closure. If surrounding tissue is limited, a free flap is generally required to close the amputation defect. This approach is acceptable and widely used, however, it can cause donor site morbidity and associated complications [4].

One way to reduce donor site morbidity is to source flap tissue from the amputated limb itself—the so-called “spare-parts” method used to fill large, complex defects resulting from trauma or tumor resection [5]. The “fillet flap” is common in spare-parts surgery, and is defined as a composite axial flap that can provide skin, muscle, fascia, and bone [5]. Fillet flaps can be pedicled or free flaps. Fillet flaps are described more often in the literature on lower limb amputation than upper limb amputation [5,6].

A review of the literature found a limited number of cases reporting fillet flaps in below-elbow amputations, and very few cases reporting the use of hand tissue for amputation defect closure. No cases were identified employing a pedicled hand fillet flap. The case presented in this report demonstrates the use of a pedicled fillet flap derived from hand tissue to close a below-elbow amputation defect where maximal stump length preservation was prioritized.

## Case report

**Figure 1.**
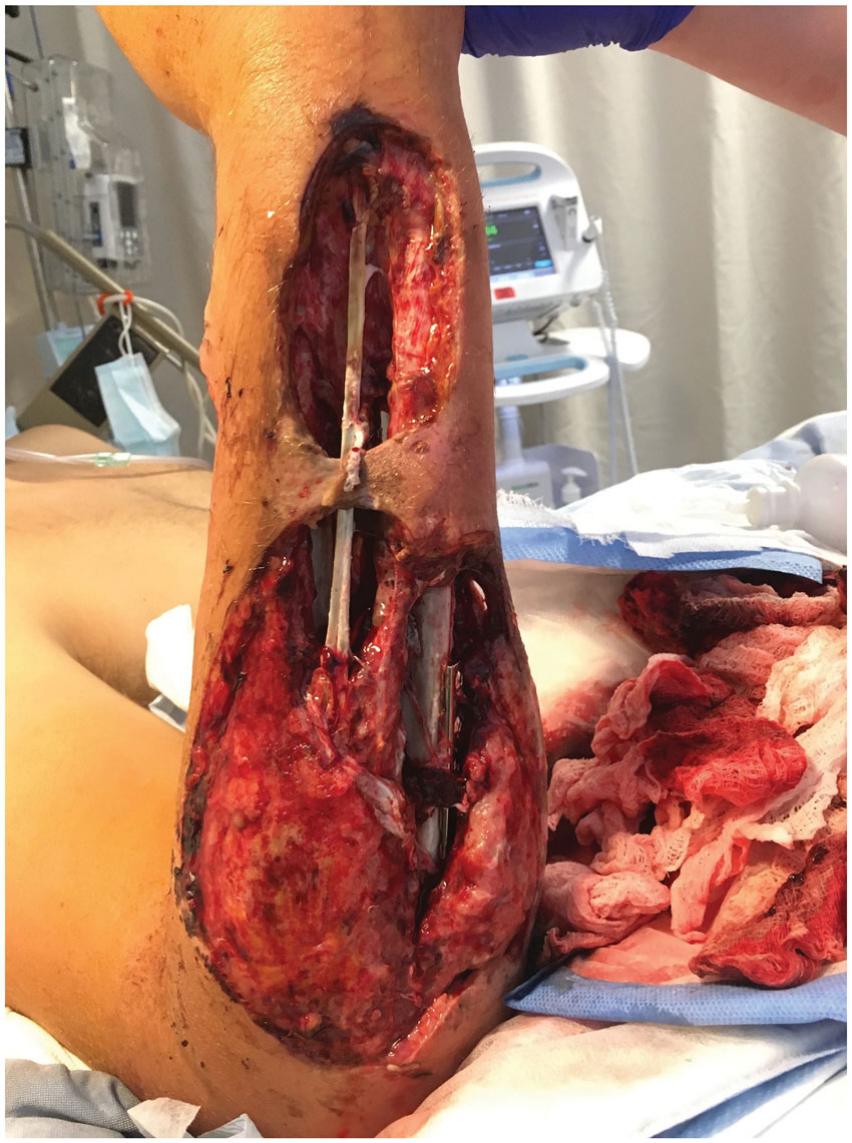
Dorsal aspect of right forearm, 9 days post-injury.

**Figure 2.**
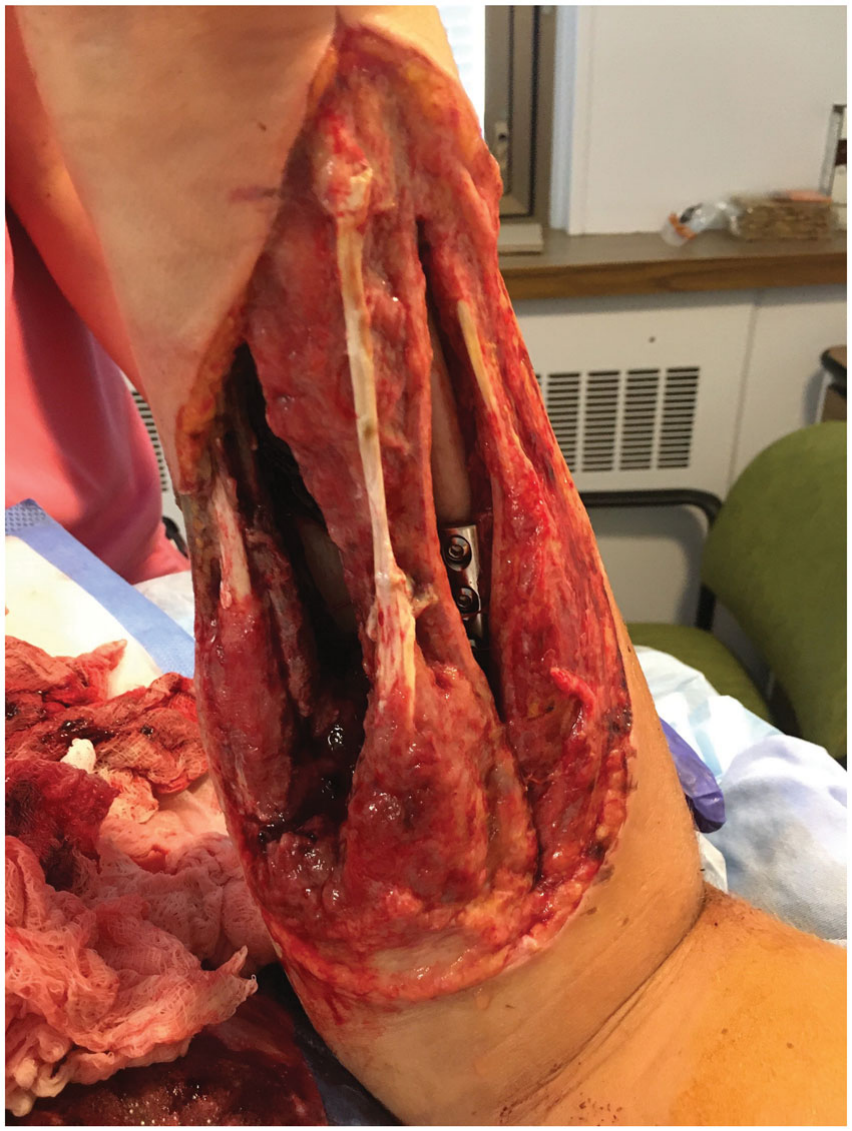
Volar aspect of right forearm, 9 days post-injury.

**Figure 3.**
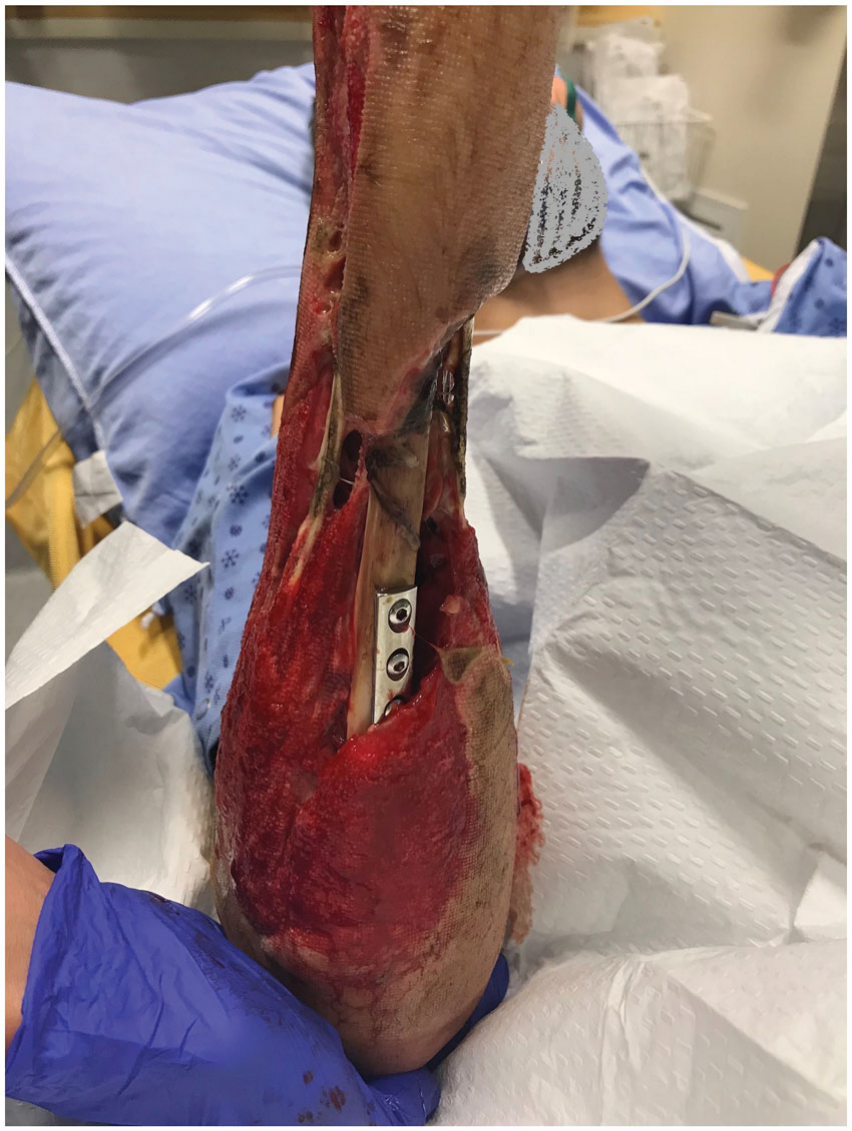
Ulnar aspect of right forearm, 17 days post-injury.

A 23-year-old male victim of a motorbike accident sustained compound proximal transverse fractures to the right radius and ulna, and a brachial plexus injury with a dense sensorimotor deficit from the shoulder distally, confirmed by MRI and EMG studies. He underwent irrigation and debridement and open reduction internal fixation of the fractures at a different hospital. Two days later, he developed progressive pain and foul incisional drainage and went for incision and drainage (I & D). Because of concerns regarding the viability of the limb he was transferred to our centre. A repeated I & D found a necrotizing infection with extensive myonecrosis in all compartments, both volar and dorsal. Microbiology of the forearm tissue showed growth of mixed gram-positive and gram-negative organisms, suggestive of a polymicrobial infection. The patient was covered with a broad-spectrum antibiotic and remained medically stable. Over the following nine days, the patient was taken to the OR six times for irrigation and debridement of the forearm. Widespread necrosis of the flexor and extensor musculature rendered two large tissue defects covering the volar and dorsal aspects of the forearm, and exposing the bones and hardware in the forearm (Figures 1–3). There was little remaining forearm flexor or extensor musculature, and the limb remained paretic and insensate, but the radial artery remained intact perfusing the hand and soft tissues. It was felt that the limb was unsalvageable distally. A below-elbow amputation was recommended and undertaken 22 days post-injury. The prolonged timeline to amputation was due to the process of having multiple consultations from three plastic and reconstructive surgeons, neurosurgery and physiatry, as well as the patient’s acceptance of his diagnosis and willingness to proceed with amputation. Although he lacked elbow flexion and extension, the prognosis for his brachial plexus injury remained unknown. Input from physiatry suggested that regeneration of the nerves may have been possible with significant rehabilitation. However, with such extensive loss of forearm musculature, regaining function of the hand would not have been possible, and recovery of mobility at the elbow was unknown. The patient hoped to someday have a below elbow prosthesis despite the guarded prognosis for nerve recovery, and so would only accept an amputation at that level. Given the amount of soft tissue loss, maintaining adequate bone stock for a prosthesis would require soft tissue augmentation for coverage of the stump. This was done by filleting the hand still perfused distally on the intact radial artery.

## Methods

**Figure 4.**
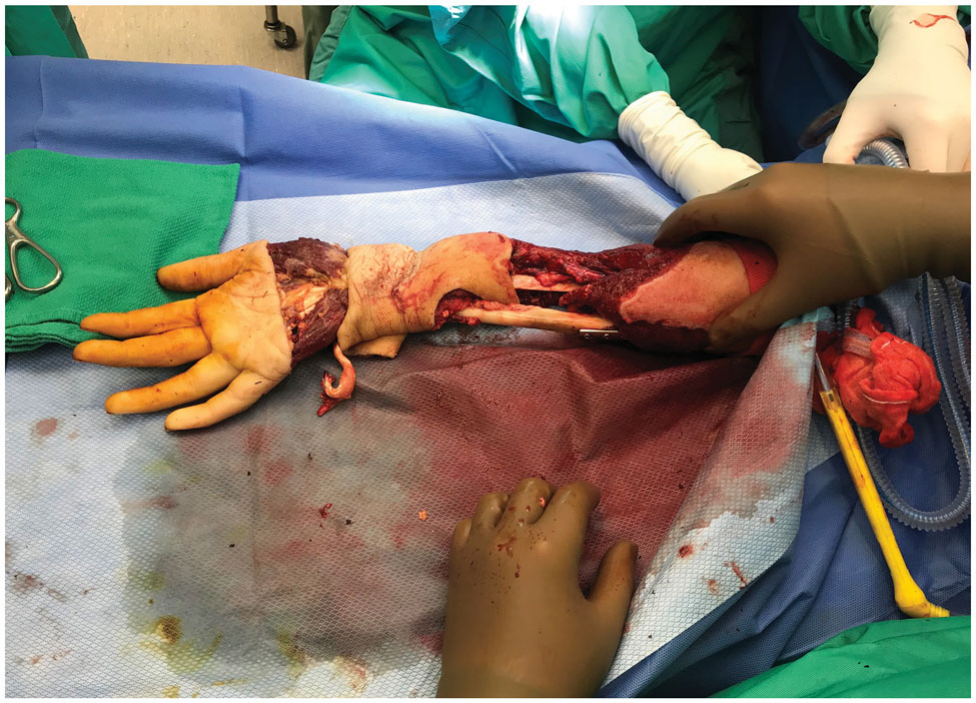
Operative method showing incision across right metacarpal heads, and filleting of hand and wrist tissue.

**Figure 5.**
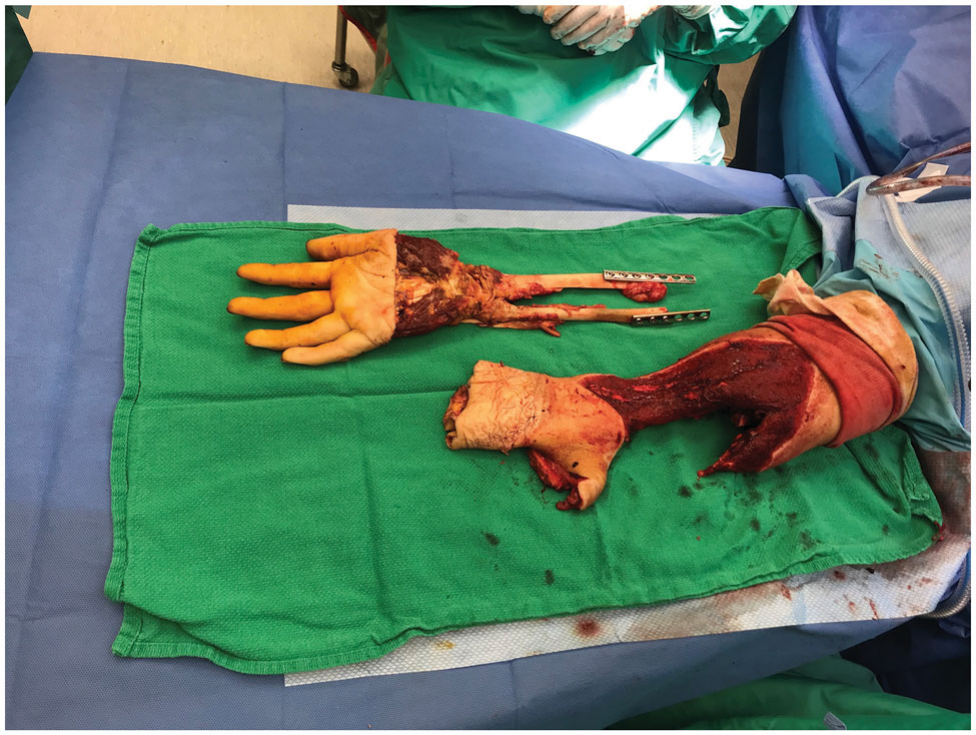
Operative method demonstrating removal of the amputated part from the right forearm, and remaining pedicled fillet flap and stump defect.

**Figure 6.**
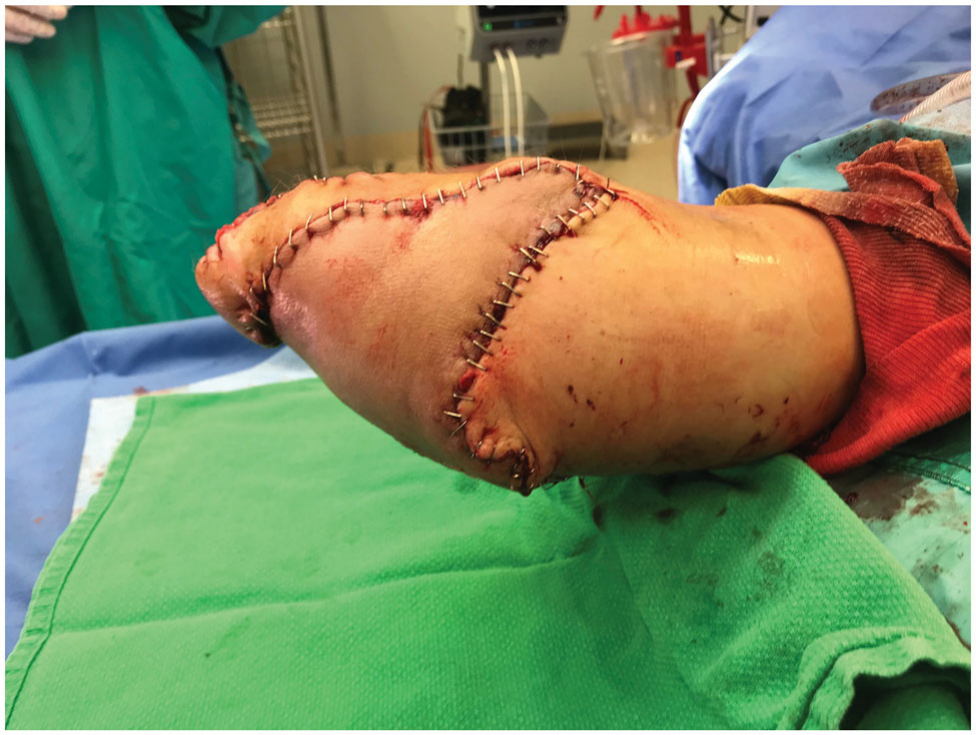
Operative method demonstrating fillet flap closure over stump defect.

Once in the OR, the right hand was assessed and deemed appropriate for harvesting a pedicled flap to cover the amputation defect. The hand was incised over the metacarpal bones, around the distal palm and the glabrous skin, and was filleted at the depth of the interosseous and intrinsic musculature and metacarpals. This technique was carried out proximally over the distal radius and ulna, such that the skeleton hand distally was filleted away from the soft tissue envelope at the metacarpal and wrist level (Figure 4). The muscle bellies of the extensor-flexor tendons that had ruptured were debrided and rotated proximally to provide soft-tissue coverage at the amputation site. The plate and screw fixation was removed from the radius and ulna in the proximal third of the forearm, and the amputated part was removed (Figure 5). The proximal bone ends were revised with a reciprocating saw. A bone stump was maintained with more than 4 cm length, as recommended by the consulting physiatrist. The tourniquet was deflated, confirming adequate perfusion of the soft tissue envelope and fillet flap distally. The neurovascular flap—based on the residual radial artery, venae comitantes, and the attached median nerve—was then rotated and reflected proximally, and seated over the deep musculature. The median nerve was included in the flap to decrease chances of neuroma formation, and to allow the potential for sensory recovery in the event of nerve regeneration. The flap was then contoured to cover the skin deficits of the amputation stump, maintaining the residual narrow radial skin bridge to the flap to keep any associated superficial venous drainage intact. The skin that had constituted the dorsum of the hand was split and the tissues reflected over the terminus of the stump on the dorsal aspect (Figure 6). There was a small 2–3 cm residual full-thickness defect just distal to the elbow on the dorsum of the forearm. Some of the trimmings from the fillet flap were defatted and used to graft the defect. There appeared to be good perfusion of the skin flap. Thereafter, a negative pressure dressing was applied to the incisions and skin graft.

## Discussion

This case describes a pedicled fillet flap derived from spare-parts hand tissue to cover a below-elbow forearm amputation following traumatic fracture and subsequent severe ischemic and septic tissue necrosis. Morris et al. state that a fish-mouth incision with volar and dorsal flaps is the ideal approach for forearm amputation [7]. However, this ideal approach is not always attainable in the setting of trauma. Following the principles laid out by Baccarani et al., when local tissue is not available for primary closure and preservation of stump length is indicated, then a spare-parts fillet flap should be used [8]. Pedicled flaps are preferred over free flaps [8]. If a fillet flap is not possible, then a traditional free or distant pedicled flap can be employed [8].

The use of free fillet hand flaps in forearm amputation defect closure is uncommon. Cavadas and Raimondi, as well as Rohrich, Ehrlichman and May describe a total of four cases in which hand free flaps provided coverage of below-elbow amputation stumps [9,10]. None of these cases employed a pedicled flap, as each case involved complete traumatic amputation at the level of the forearm.

In this case, amputation was indicated due to a non-reconstructible soft-tissue defect caused by extensive myonecrosis of the flexor and extensor musculature. Viable and well-perfused tissue from the right hand was available for pedicled fillet flap coverage of the amputation defect. These factors supported proceeding with spare-parts surgery. However, alternative approaches were considered prior to proceeding with amputation, including reconstruction, above-elbow amputation, and below-elbow amputation with traditional flap closure. Consultations from a total of three hand and upper limb surgeons, as well as physiatry and neurosurgery, determined there was no way to salvage any functionality of the arm distal to the elbow, and potentially not proximal to it. Reconstruction of the forearm soft-tissue defect alone would not have provided any benefit and would have contributed to greater risk and morbidity. A traditional flap was contraindicated given the availability of a pedicled fillet flap. Had the hand fillet flap not fully covered the defect, a traditional flap may have been considered to cover the remaining defects. Skin grafting was not considered for stump coverage, as split-thickness skin grafts can develop ulceration or overt wound failure in the context of direct pressure and shear forces from regular prosthesis wear [7]. Coverage with vascularized tissue is preferred [11]. Lastly, while above-elbow amputation, in theory, may have been an acceptable approach given the associated brachial plexopathy and lack of elbow flexion and extension, the patient’s preference was to proceed with below-elbow amputation in hopes of maintaining the potential for a below-elbow prosthesis where he ever to regain motor function at the elbow.

**Figure 7.**
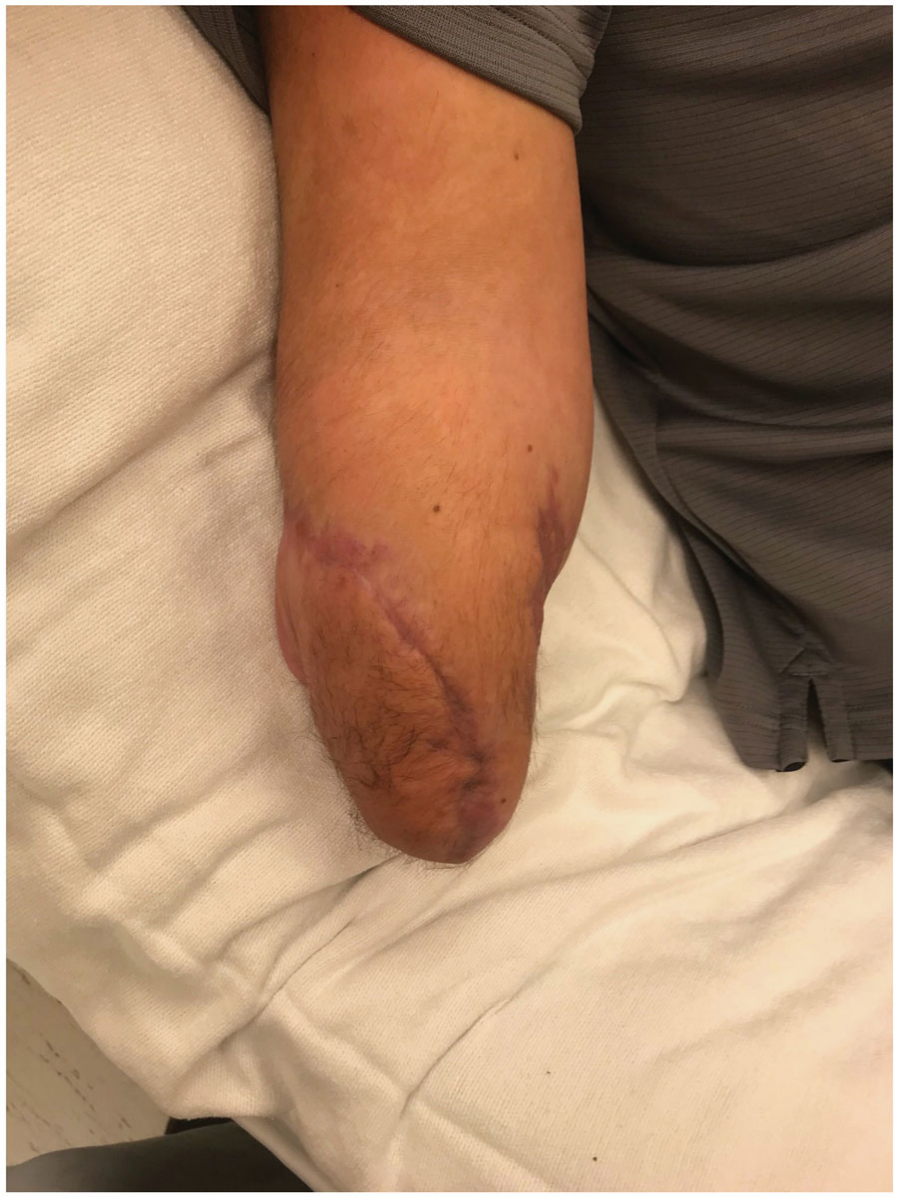
Follow up at post-op week 10 showing excellent wound healing and coverage of the stump defect, dorsoradial aspect.

**Figure 8.**
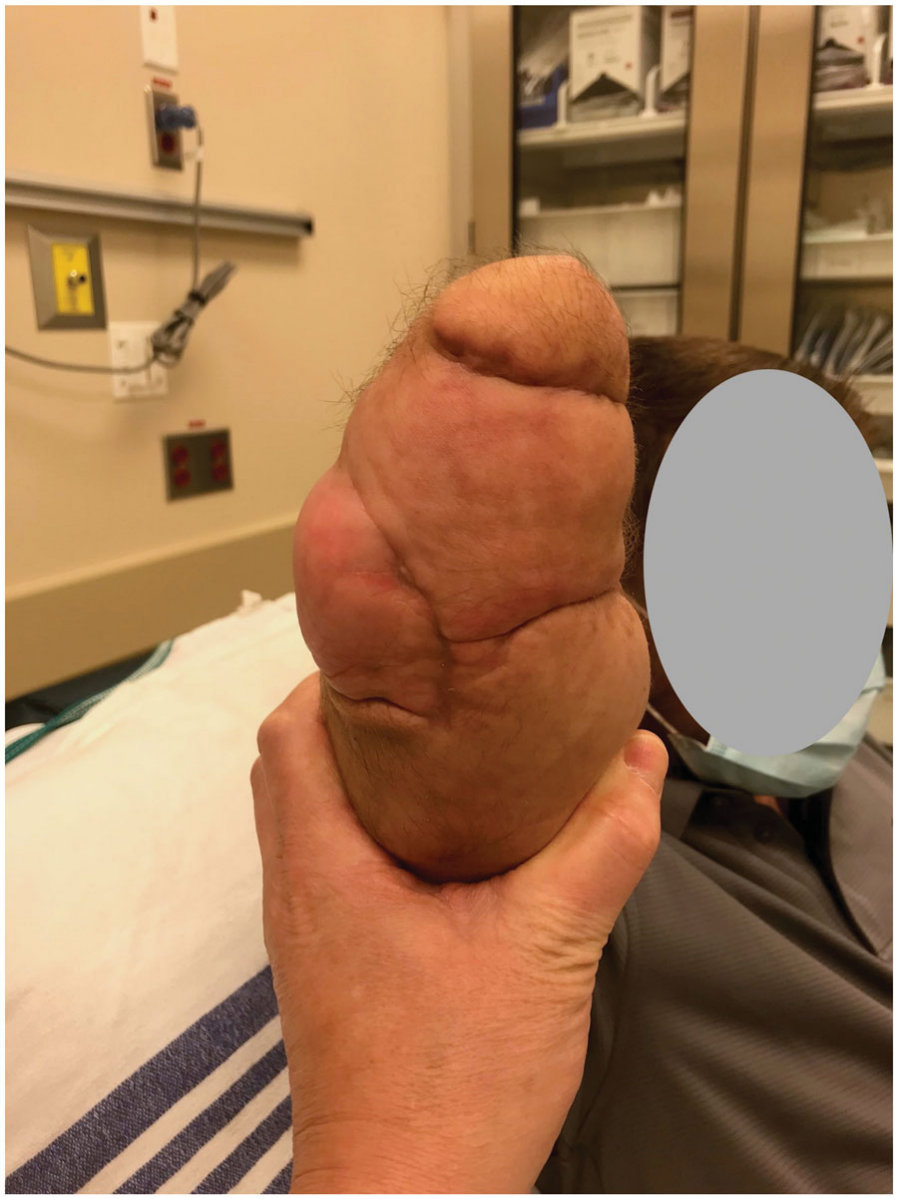
Follow up at post-op week 10 showing excellent wound healing and coverage of the stump defect, volar aspect.

In the immediate postoperative week, the stump wounds required two additional minor debridements due to ongoing infection. However, there were no further complications and the fillet flap covering the stump remained healthy at 10 weeks post-op (Figure 7, 8). At this time, the patient had regained shoulder motion, however, he had little recovery of motion at the elbow.

## Conclusion

The case reported here demonstrates that spare-parts surgery is an effective and preferred approach in forearm amputation where length preservation is prioritized but tissue for closure is lacking. Hand tissue can be used as a pedicled fillet flap for amputation defect closure when adequate perfusion to the hand remains intact.
